# Pulmonary Veno-Occlusive Disease in Rheumatoid Arthritis: A Rare Pathological Entity Independent of Interstitial Lung Disease

**DOI:** 10.3390/diagnostics16030382

**Published:** 2026-01-24

**Authors:** Rina Izumi, Koji Hayashi, Ei Kawahara, Yuka Nakaya, Asuka Suzuki, Mamiko Sato, Naoko Takaku, Toyoaki Miura, Hiromi Hayashi, Kouji Hayashi, Yasutaka Kobayashi

**Affiliations:** 1 Department of Rehabilitation Medicine, Fukui General Hospital, 55-16-1 Egami-cho, Fukui 910-8561, Japan; 2 Department of Pathology, Fukui General Hospital, 55-16-1 Egami-cho, Fukui 910-8561, Japan; 3 Graduate School of Health Science, Fukui Health Science University, 55-13-1 Egami, Fukui 910-3190, Japan; 4 Department of Neurology, University of Fukui Hospital, 23-3 Matsuoka Shimoaizuki, Eiheiji-cho, Yoshida-gun, Fukui 910-1193, Japan

**Keywords:** pulmonary veno-occlusive disease, rheumatoid arthritis, elderly, autopsy, pathology, case report, interstitial lung disease

## Abstract

We present the case of an 83-year-old woman with a long-standing history of rheumatoid arthritis (RA) who was found collapsed at home. The patient presented with cardiopulmonary arrest and could not be resuscitated. A postmortem examination was performed to determine the cause of death. Postmortem computed tomography (CT) ruled out intracranial hemorrhage but revealed diffuse bilateral pulmonary consolidations and signs of bronchial obstruction. The autopsy revealed severe pulmonary edema and marked right ventricular hypertrophy. Microscopic examination of the lungs demonstrated characteristic features of pulmonary veno-occlusive disease (PVOD), including widespread fibrous intimal thickening and occlusion of small pulmonary veins and venules. Notably, there was no evidence of RA-associated interstitial lung disease (ILD). The direct cause of death was identified as pulmonary edema secondary to PVOD. This case highlights that PVOD can occur in patients with RA as a distinct pathological entity, independent of ILD. This finding is significant as it contrasts with previous reports where PVOD was associated with ILD. Therefore, clinicians should consider PVOD in the differential diagnosis of RA patients who present with unexplained pulmonary hypertension or progressive dyspnea, even in the absence of interstitial lung disease.

**Figure 1:**
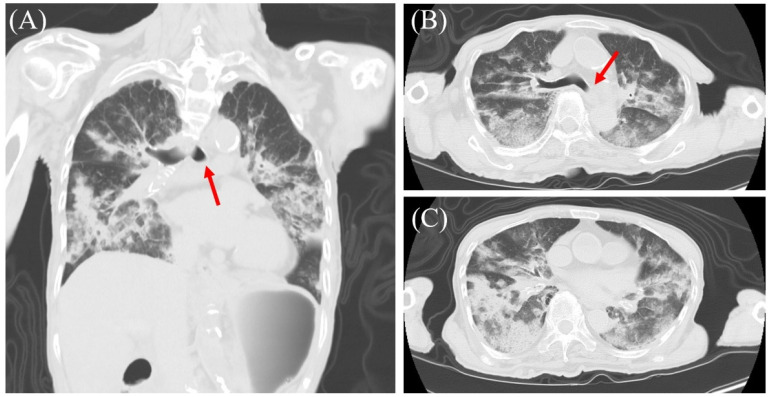
Autopsy imaging results obtained using computed tomography (CT). (**A**–**C**) CT scans showing obstruction of the left main bronchus (arrows) and bilateral consolidations consistent with severe pulmonary edema over large areas of both lung fields. The patient was an 83-year-old woman who found collapsed at home by her family. She had a history of diabetes mellitus, dyslipidemia, Alzheimer’s disease, post-operative colon cancer, cataracts, and a rib fracture. She also had a history of rheumatoid arthritis (RA), diagnosed at age 71, for which she was treated with methotrexate at age 78. There was no clinical history of chronic heart failure, valvular heart disease, or chronic obstructive pulmonary disease (COPD). Echocardiography performed 9 years prior showed a preserved ejection fraction (69%) with only mild mitral and tricuspid regurgitation. However, she discontinued her oral medication and stopped attending appointments based on her own judgment, without consulting her doctor. She was transported to our hospital in cardiopulmonary arrest, but resuscitation efforts were unsuccessful, and death was confirmed 1 h and 19 min after her family discovered her. Autopsy imaging and a pathological autopsy were performed at the time of death.

**Figure 2:**
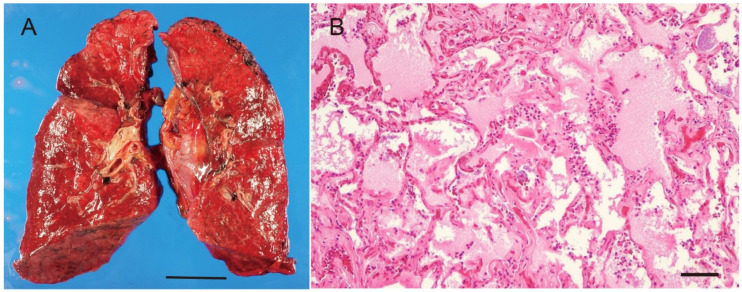
Postmortem analysis of the lungs. (**A**) Macroscopically, the lungs were voluminous (right 680 g, left 470 g) and exhibited marked edema and mild congestion with scattered small foci of bronchopneumonia. Scale bar = 5 cm. (**B**) Microscopically, alveolar edema was prominent. Scale bar = 50 µm.

**Figure 3:**
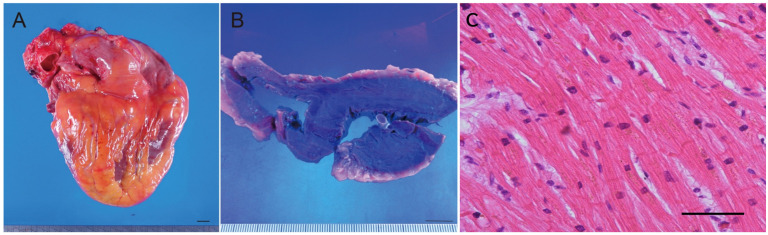
Autopsy findings of the heart. (**A**) Posterior surface of the heart. Right ventricle was hypertrophied. The heart weighed 360 g. Macroscopic examination revealed a left ventricular posterior wall thickness of 14 mm and a right ventricular posterior wall thickness of 9 mm. (**B**) Cut surface of the heart showing marked hypertrophy of the wall of the right ventricle and mild hypertrophy of the left ventricle. Scale bar = 1 cm. (**C**) High-power microscopic view of the right ventricular wall (Hematoxylin and Eosin stain, original magnification ×400). Morphometric analysis confirmed pathological hypertrophy, with cardiomyocytes showing a mean transverse diameter of 21.4 µm. These findings strongly suggest the presence of severe, chronic pulmonary hypertension. Scale bars = 1 cm (**A**), 50 µm (**B**).

**Figure 4:**
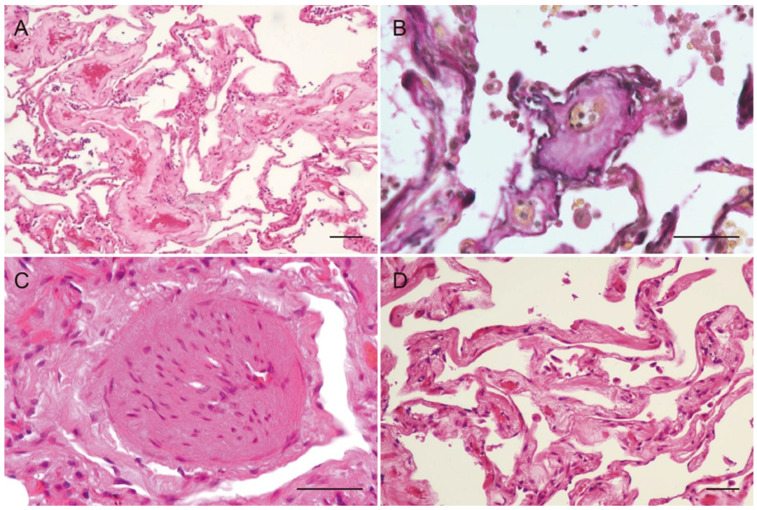
Microscopic findings of pulmonary veno-occlusive disease (PVOD). (**A**) Small veins, venules, and capillaries showed thickening of the vascular walls, luminal narrowing with markedly hyalinized intima. (**B**) Small veins with an outer elastic lamina, demonstrated by elastic van Gieson stain, revealed severe luminal narrowing and pronounced hyalinization of the intima and media. (**C**) Small arteries displayed marked hypertrophy of the media, suggesting pulmonary hypertension. (**D**) Mild focal fibrosis was observed, particularly surrounding small veins and venules, suggesting these are associated with long-standing PVOD. Scale bars = 50 µm. PVOD is a very rare condition characterized by the obstruction or narrowing of the pulmonary veins and small venules, leading to pulmonary hypertension [1,2,3]. Patients with PVOD exhibit a bimodal age distribution, with the first peak observed in the 20–30 years age range and a second peak occurring in the 70–80 years age range [4]. Uncommon types of pulmonary arterial hypertension (PAH) characterized by remodeling of the pulmonary veins and capillaries are classified as PAH associated with signs of venous and/or capillary involvement, specifically PVOD and pulmonary capillary hemangiomatosis (PCH) [1,2,3]. PVOD and PAH exhibit similar clinical signs, including progressive dyspnea, fatigue, and exercise intolerance [1]. However, patients with PVOD typically present with a more severe phenotype, characterized by pronounced dyspnea and hypoxemia [5,6]. Early diagnosis of PVOD is crucial, as these patients often have a poor response to PAH-specific therapies and face an increased risk of pulmonary edema, along with a worse prognosis without lung transplantation compared to other forms of PAH [7]. RA is an autoimmune disease that primarily affects the joints but can also lead to various pulmonary complications. In particular, interstitial lung disease (ILD) is a common pulmonary complication in patients with RA and can result in significant morbidity and mortality [8]. As far as we know, there are limited published studies documenting the association between RA and PVOD [9]. The obstruction of the left main bronchus observed on CT was considered a terminal event, possibly due to aspiration or mucus plugging, contributing to the acute respiratory failure, rather than a primary feature of PVOD. The histopathological findings were consistent with PVOD, characterized by marked intimal thickening, stenosis, and occlusion of small pulmonary veins. Fibrosis of the alveolar septa, pulmonary edema, and right ventricular hypertrophy were also observed. No evidence of RA-associated ILD or PCH was found. Crucially, a thorough pathological evaluation revealed no histological patterns indicative of RA-associated interstitial lung disease, such as usual interstitial pneumonia or non-specific interstitial pneumonia. Additionally, there was no evidence of capillary proliferation or congestion suggestive of pulmonary capillary hemangiomatosis. RA is associated with various pulmonary complications, including ILD, airway disease, pleural disease, and vascular involvement [8]. ILD is a well-recognized manifestation of RA, most commonly presenting as UIP or NSIP [8]. In addition, RA can lead to pulmonary hypertension, vasculitis, and Caplan syndrome [8]. However, the relationship between RA and PVOD remains unclear. The exact causes and risk factors for PVOD remain unclear; however, suggested risk factors include genetic mutations (EIF2AK4) [6], smoking, chemotherapy, and connective tissue diseases [4]. Among connective tissue diseases, PVOD has been reported in cases of Sjögren’s syndrome [10,11], mixed connective tissue disease (MCTD) [12], Felty’s syndrome [13], and adult-onset Still’s disease [14], as well as in one previously reported RA case [9]. The previously reported RA-associated PVOD case demonstrated honeycomb changes in the lower lobes and fibrotic changes in the upper and middle lobes, findings consistent with RA-associated ILD [9]. In addition, widespread capillary proliferation was observed, suggesting the presence of PCH [9]. In contrast, no RA-associated ILD or PCH was observed in the present case. The predominant pathological findings were venous remodeling and occlusion, with minimal arterial involvement. This distinction suggests that PVOD in RA patients may manifest through different pathological pathways, potentially independent of ILD. This case suggests that PVOD in RA patients may develop independently of ILD. Although previous reports have described PVOD and PCH in RA patients with ILD [9], the precise mechanisms underlying their development remain unclear. ILD-associated hypoxia and chronic inflammation have been proposed as potential contributing factors, but their roles have not been definitively established. It is also possible that PVOD developed independently of RA in this case. However, since connective tissue diseases, including RA, have been implicated in PVOD pathogenesis, this case may represent a distinct form of RA-associated PVOD. Although the patient was 83 years old, falling into the second peak of the bimodal age distribution for sporadic PVOD, the long-standing history of RA suggests a potential pathogenic link. Given the absence of other risk factors such as chemotherapy or organic solvent exposure, we consider this case to be RA-associated PVOD. Further studies are required to elucidate the underlying mechanisms of PVOD in RA and to improve diagnostic strategies.

## Data Availability

The data presented in this study are available on request from the corresponding author due to patient privacy and ethical considerations.
